# Integrated Prognostic Modelling with the CALLY Index and Interim PET/CT in Hodgkin Lymphoma

**DOI:** 10.3390/cancers18193205

**Published:** 2026-10-04

**Authors:** Hasan Göze, İsmail Eren Polat, Nermin Bal, İsa Yalçınkaya, Gokhan Burul, Büşra Tuğçe Tonyalı, Tahir Alper Cinli, İstemi Serin

**Affiliations:** Department of Hematology, Başakşehir Çam and Sakura City Hospital, 34480 Istanbul, Turkey

**Keywords:** Hodgkin lymphoma, CALLY index, interim PET/CT, Deauville score, inflammation, prognosis

## Abstract

**Background/Objectives**: Interim ^18^F-FDG PET/CT is the most powerful prognostic tool in Hodgkin lymphoma but is only available after 2 cycles and does not guide initial treatment. The C-reactive protein-albumin-lymphocyte (CALLY) index is a novel composite index of inflammation, nutrition, and immunity, measurable at diagnosis, but has not been studied in Hodgkin lymphoma (HL). We tested whether baseline CALLY could predict interim PET response and survival and whether combining the two provides additional information over either alone. **Methods**: A retrospective study of 180 consecutive patients with newly diagnosed Hodgkin lymphoma at a single center was performed. CALLY was defined as albumin (g/L) × lymphocytes (×109/L)/CRP (mg/L). Interim PET after two cycles was scored using the Deauville scale (4–5 positive). The endpoints were progression-free survival (PFS) and overall survival (OS). **Results**: Median follow-up was 30.5 months, 32 patients had a PFS event, and 16 died. Interim PET was positive in 31/163 patients (19.0%). Baseline CALLY was lower in PET-positive patients compared with PET-negative patients (median 1.16 vs. 3.34; *p* = 0.022; area under the curve 0.635, modest discrimination). Interim PET positivity (29.8% vs. 13.0%; OR 2.84, 95% CI 1.26–6.42), 24-month PFS (66.9% vs. 89.8%; *p* = 0.001) and OS (84.8% vs. 95.5%; *p* = 0.022) were reduced below a Youden-derived threshold of 1.5. CALLY was not independently prognostic after adjustment for interim PET (PFS hazard ratio [HR] 1.86, 95% CI 0.79–4.37; *p* = 0.157). A three-tier exploratory model combining both factors separated 24-month PFS into 94.5% (*n* = 87), 79.6% (n = 53), and 41.2% (n = 17) (*p* < 0.001; high- vs. low-risk HR 9.48, 95% CI 3.59–25.04), although the intermediate tier was not statistically different from the low-risk tier. **Conclusions**: In this cohort, baseline CALLY provides a low-cost pre-treatment correlate of early metabolic response and outcome, with numerically greater discrimination than the International Prognostic Score. Its prognostic signal seems largely mediated through the interim PET response, and therefore it should complement, not replace, interim response assessment. Discrimination was modest, and the integrated model is exploratory and requires prospective multicenter validation in contemporary protocols before clinical use.

## Simple Summary

Hodgkin lymphoma is a highly curable lymphoid malignancy; however, approximately one in five patients does not respond adequately to initial treatment. A scan performed after the first two treatment cycles is currently the best way to find these patients, yet it comes too late to guide the choice of initial therapy. We tested whether a simple score calculated from three routine blood tests taken at diagnosis, reflecting inflammation, nutrition, and immune status, could identify them earlier. Among 180 patients treated at our hospital, those with a low score were more than twice as likely to show a poor response on the early scan and were more likely to experience disease progression. However, the score added little once the early scan result was known, which suggests that it works mainly by predicting how the lymphoma responds to treatment. Combining the score with the scan divided patients into three groups with different outcomes, but this grouping is exploratory: the worst group contained only 17 patients. Because these blood tests are already performed for every patient at no extra cost, the score could help doctors plan closer monitoring from the first day, although it must be confirmed in other patient groups before it is used in routine care.

## 1. Introduction

Roughly one in five patients with advanced-stage Hodgkin lymphoma relapses or progresses after first-line therapy [1], whereas the majority are cured but remain exposed to anthracyclines and bleomycin, at the cost of a measurable long-term excess of pulmonary disease [2]. Prognostication in this disease therefore has to work in two directions at once: identifying the minority who need intensification and sparing the majority who do not.

Two families of tools dominate practice. The first comprises scores assigned at diagnosis, the prime example being the International Prognostic Score (IPS) of Hasenclever and Diehl [3]. It was derived before response-adapted therapy, and it adds little once early response is known [4]. Inflammation-based indices proposed alongside it have contributed little independent information [5]. The second is response adaptation based on interim ^18^F-FDG PET/CT after two cycles, read according to the Deauville five-point scale [6,7]. Interim PET has consistently outperformed baseline scores [4,8] and has formed the basis of randomized response-adapted strategies, including RATHL and AHL2011 [9,10].

The practical limitation of interim PET is timing. It is informative precisely because it shows how the disease behaves under therapy, which also means that it cannot inform the initial treatment decision. If a marker predicting interim response could be identified before treatment, clinicians could flag patients for closer monitoring, earlier imaging, or trial enrollment before the first cycle is given.

The tumor microenvironment is a plausible source of such a marker. In classical Hodgkin lymphoma, the malignant Hodgkin and Reed–Sternberg cells typically account for less than 1% of the cells in a tumor biopsy and are vastly outnumbered by the surrounding reactive infiltrate [11]; the composition of that infiltrate correlates with outcome [12,13]. The infiltrate is sustained by cytokines such as interleukin-6, whose circulating levels independently predict treatment failure [14]. Three routine laboratory values can act as systemic surrogates of this process: an elevated C-reactive protein (CRP), hypoalbuminemia, and lymphopenia. Each has been associated with disease extent or treatment response in Hodgkin lymphoma—CRP [15] and lymphopenia [16] directly, and albumin and lymphocyte count as components of the IPS [3]. More broadly, cancer-related systemic inflammation is linked to reduced treatment effectiveness [17].

The C-reactive protein–albumin–lymphocyte (CALLY) index integrates these three variables into a single continuous value. It was first described by Iida et al. in resected hepatocellular carcinoma [18]; its prognostic value has since been supported by a systematic review and meta-analysis across solid tumors [19] and, more recently, by a study in diffuse large B-cell lymphoma [20]. To our knowledge, it has not been studied in Hodgkin lymphoma and has never been compared with interim PET response in any lymphoma.

We therefore set out to answer three questions in a single-center cohort of patients with newly diagnosed Hodgkin lymphoma: whether baseline CALLY is associated with interim PET positivity; whether it predicts PFS and OS; and whether an integrated model combining CALLY with interim PET discriminates better than either component alone or than the IPS.

## 2. Patients and Methods

### 2.1. Study Design and Population

We retrospectively identified, from the institutional database, consecutive patients aged ≥18 years with histologically confirmed Hodgkin lymphoma who were diagnosed and treated at a single tertiary hematology center. Of 183 screened records, 2 lacked a diagnosis date, last follow-up date, or vital status, and 1 had a non-positive follow-up interval, leaving 180 patients for analysis (Supplementary Figure S1). 

The study is reported in accordance with the Strengthening the Reporting of Observational Studies in Epidemiology (STROBE) statement [21]; the completed checklist is provided as Supplementary Materials File S1. The study was conducted in accordance with the Declaration of Helsinki and was approved by the institutional ethics committee (approval no. 373; 2 July 2026). The requirement for individual informed consent was waived because of the retrospective design and the use of de-identified data.

### 2.2. Staging, Treatment and Response Assessment

Staging was performed according to the Ann Arbor system with the Cotswolds modifications [22], based on baseline ^18^F-FDG PET/CT. Bulky disease was defined, following the same criteria [22], as a nodal mass ≥ 10 cm or a mediastinal mass ratio > 0.33, and bone marrow involvement was recorded from PET/CT and, when performed, from biopsy. First-line treatment followed institutional practice and consisted mainly of ABVD (doxorubicin, bleomycin, vinblastine and dacarbazine), with brentuximab vedotin–AVD [23] in selected patients with advanced-stage disease and radiotherapy at the discretion of the treating team.

Interim PET/CT was performed after two cycles of chemotherapy. Scans were reported by nuclear medicine physicians using the Deauville five-point scale [6]; scores of 1–3 were classified as negative and 4–5 as positive, in line with the Lugano classification [7]. End-of-treatment PET/CT was classified, by the same criteria, as complete metabolic response (Deauville 1–3) or inadequate response (Deauville 4–5).

### 2.3. The CALLY Index and Comparator Markers

Peripheral blood counts and biochemistry obtained at diagnosis, before any therapy, were used. The CALLY index was calculated as:

$$\mathrm{CALLY}=\frac{\mathrm{albumin}\;(g/L)\times \mathrm{absolute}\;\mathrm{lymphocyte}\;\mathrm{count}\;(\times \;10^{9}/L)}{\mathrm{CRP}\;(\mathrm{mg}/L)}$$

The index was originally proposed in resected hepatocellular carcinoma by Iida et al. [18]. Because the only previous application in lymphoma is the most relevant comparator for our values, we used the scale of Cvetković et al. in diffuse large B-cell lymphoma [20]. Laboratory values recorded in other units were converted before calculation: albumin from g/dL to g/L and lymphocytes from cells/µL to ×10^9^/L. Higher CALLY values indicate a more favorable inflammatory, nutritional, and immune profile. As a worked example, a patient with an albumin of 4.0 g/dL, an absolute lymphocyte count of 1600/µL and a CRP of 30 mg/L has an albumin of 40 g/L and a lymphocyte count of 1.6 × 10^9^/L, giving CALLY = (40 × 1.6)/30 = 2.13. Throughout this manuscript, laboratory values are tabulated in the units used in clinical reports (albumin g/dL, lymphocytes /µL, CRP mg/L), whereas the index is computed in the units of the formula above; to reproduce our values from Table 1 or Table 2, multiply albumin by 10 and divide the lymphocyte count by 1000.

Two scales are in use for this index, and the distinction matters when thresholds are compared across studies. In the original report and in most of the subsequent surgical oncology literature, albumin (g/dL) is multiplied by lymphocytes (cells/µL) and divided by CRP (mg/dL) × 10^4^ [18], which yields values exactly one-tenth of those reported here. Cvetković et al. [20] print the 10^4^ divisor together with units of g/L and ×10^9^/L; with those units, virtually every value would fall several orders of magnitude below their stated cut-off of 6.5, so the scale their results actually describe is the one without the divisor, and this is the scale we have matched. Because the two conventions differ only by a constant factor, they rank patients identically: every area under the curve, hazard ratio, correlation coefficient, and *p* value reported here is unaffected by the choice, and only the numerical value of a threshold changes. Readers using the g/dL convention should divide our cut-off by 10.

For comparison, we calculated the neutrophil-to-lymphocyte ratio (NLR), the platelet-to-lymphocyte ratio (PLR), the lymphocyte-to-monocyte ratio (LMR), and the seven-factor IPS [3], the latter dichotomized at the conventional threshold of ≥3. In an exploratory analysis, the same CALLY formula was applied to laboratory values obtained at the time of interim PET (Supplemantary Materials Table S5).

### 2.4. Endpoints

Progression-free survival (PFS) was defined as the time from diagnosis to the first documented disease progression or relapse, or death from any cause. Overall survival (OS) was defined as the time from diagnosis to death from any cause. Median follow-up was estimated with the reverse Kaplan–Meier method [24].

### 2.5. Statistical Analysis

Continuous variables are reported as median (interquartile range) and compared by the Mann–Whitney U test; categorical variables are reported as counts (percentages) and compared by Fisher’s exact test. All tests were two-sided with α = 0.05.

The ability of CALLY to discriminate interim PET positivity was assessed with the area under the receiver operating characteristic curve (AUC), with 95% confidence intervals derived from 2000 bootstrap resamples.

Derivation of the CALLY threshold: No threshold for this index has been established in Hodgkin lymphoma, and values published in other diseases are not transferable to a population with this inflammatory burden; a cut-off was therefore derived from the present data, using a single endpoint, interim PET positivity. Across the receiver operating characteristic curve for CALLY against the interim PET result, we computed the Youden index (sensitivity + specificity − 1) [25] at every observed value of the index and selected the value that maximized it. The maximum occurred at CALLY = 1.51 (Youden J = 0.285). We rounded this down to 1.5, because two significant figures reflect the precision the scale supports and a round value is easier to apply at the bedside. Rounding moved one patient from the low-CALLY to the high-CALLY group, so the applied rule has a slightly lower sensitivity than the unrounded optimum; the operating characteristics reported below are those of the rule as applied. The rounded value was then used unchanged in every subsequent analysis and was not re-optimized for any other endpoint.

Deriving the threshold from interim PET rather than from survival was deliberate. A cut-off chosen to maximize separation of the survival curves and then tested on those same curves would be circular, and the resulting hazard ratios and log-rank *p* values would be optimistic by construction [26]. Anchoring the cut-off to an endpoint measured independently of survival removes that particular bias from the progression-free and overall survival analyses, although the threshold remains data-derived and its optimism is addressed below.

The stability of the threshold was examined in three ways. First, cut-off selection was repeated in 2000 bootstrap resamples to describe the sampling distribution of the Youden-optimal value. Second, survival separation was re-estimated across a grid of alternative thresholds spanning the plausible range, to establish whether the finding depends on the particular value chosen. Third, the internal validation described below applied optimism correction to every model that used the dichotomized variable. Results of the first two checks are given in Supplementary Table S2. Because the threshold was derived rather than prespecified, all analyses using it require external validation.

Associations with interim PET positivity were modeled with logistic regression. Survival distributions were estimated with the Kaplan–Meier method and compared with the log-rank test. Hazard ratios were estimated with Cox proportional hazards models, and the proportional hazards assumption was assessed with tests based on scaled Schoenfeld residuals [27]. Because interim PET is measured after diagnosis, we also performed a landmark analysis anchored at the interim PET date to guard against immortal-time bias [28]. Ann Arbor stage was modelled throughout as a binary variable (early, stage I–II, vs. advanced, stage III–IV) rather than as four separate categories, because only 9 patients had stage I disease, and category-specific estimates would not have been interpretable.

Model discrimination was evaluated with Harrell’s concordance index (C-index) [29]. Internal validation used 500 bootstrap replications with out-of-bag evaluation and Harrell’s optimism correction; this approach was chosen because the ordinary bootstrap distribution is degenerate for a single binary predictor. Nested models were compared with likelihood ratio tests. Time-dependent discrimination at 24 months was evaluated with a cumulative/dynamic AUC [30] (Supplemantary Materials Table S4).

Analyses used complete cases; the extent of missingness is reported in Supplementary Table S3. Statistical analyses were performed using IBM SPSS Statistics for Windows, Version 26.0 (IBM Corp., Armonk, NY, USA) and Python 3.14 (pandas, statsmodels and SciPy).

## 3. Results

### 3.1. Cohort Characteristics

The 180 patients had a median age of 34 years (IQR 25–51), and 112 (62.2%) were male; nodular sclerosis was the most common histology, 54.2% had advanced-stage disease, and 40.3% had an IPS of 3 or more (Table 1). ABVD was the most common regimen. Ten of 178 patients (5.6%) received radiotherapy, and 10 of 161 (6.2%) underwent autologous stem cell transplantation.

Median follow-up was 30.5 months (reverse Kaplan–Meier method). Thirty-two patients had a PFS event, and 16 died. Interim PET was positive in 31 of 163 evaluable patients (19.0%), and 127 of 146 (87.0%) achieved a complete metabolic response at the end of treatment. Interim PET positivity was strongly associated with both end-of-treatment response (complete response 14/28 vs. 112/117; *p* < 0.001) and subsequent relapse (13/30 vs. 9/131; *p* < 0.001), confirming the internal validity of the imaging endpoint in this cohort.

Median baseline CALLY was 2.50 (IQR 0.50–10.32).

### 3.2. CALLY and Interim PET Response

In the 157 patients with both measurements, baseline CALLY was significantly lower in those with a subsequent positive interim PET: median 1.16 (IQR 0.41–3.25) vs. 3.34 (IQR 0.80–11.34); *p* = 0.022 (Figure 1A). CALLY had an AUC of 0.635 (95% CI 0.525–0.739) for interim PET positivity, and the applied threshold of 1.5 yielded a sensitivity of 56.7% (95% CI 39.2–72.6) and a specificity of 68.5% (95% CI 60.0–75.9) (Figure 1B); the unrounded Youden point of 1.51 would have yielded 60.0% and 68.5%, respectively. An area under the curve of this magnitude represents modest discrimination: baseline CALLY separates patients who will and will not have a positive interim scan better than chance, but not with the accuracy expected of a stand-alone diagnostic or triage test.

Among the individual CALLY components and the comparator markers, only CRP was significantly associated with interim PET positivity (AUC 0.655; *p* = 0.008); NLR, IPS, albumin, LMR and PLR were not (Table 3).

Patients with CALLY < 1.5 had more than twice the rate of interim PET positivity: 17 of 57 (29.8%) vs. 13 of 100 (13.0%) (Figure 1C), corresponding to an unadjusted odds ratio of 2.84 (95% CI 1.26–6.42; *p* = 0.012). The only other significant univariable predictor was extranodal involvement (OR 2.87, 95% CI 1.29–6.38; *p* = 0.010). In a multivariable model including CALLY, advanced stage, bulky disease, and extranodal involvement (n = 157; 30 events), extranodal involvement remained significant (OR 5.09, 95% CI 1.49–17.43; *p* = 0.010), whereas the association with CALLY was attenuated to borderline significance (OR 2.38, 95% CI 0.95–5.96; *p* = 0.064). In this model, advanced stage had an inverse coefficient (OR 0.28, 95% CI 0.08–0.99; *p* = 0.048), which we interpret as model instability rather than a protective effect: the upper confidence limit approaches unity, the direction is opposite to that of the univariable estimate, and the model explained little of the overall variation (McFadden pseudo-R^2^ = 0.098).

### 3.3. CALLY and Disease Biology

Low CALLY was strongly associated with disease burden and systemic inflammation (Table 2). Patients with low CALLY had lower hemoglobin, lymphocyte count, and albumin, and markedly higher CRP (123.0 vs. 10.4 mg/L) and erythrocyte sedimentation rate (ESR). They more often presented with B symptoms, extranodal involvement, bone marrow involvement, advanced stage, and an IPS ≥ 3 (79.1% vs. 16.0%); each of these comparisons was significant at *p* < 0.001. Age and sex did not differ between the groups.

### 3.4. Survival

Patients with CALLY < 1.5 had significantly worse PFS, with 24-month estimates of 66.9% vs. 89.8% and 36-month estimates of 63.3% vs. 85.2% (log-rank *p* = 0.001; HR 3.01, 95% CI 1.48–6.14) (Figure 2A). OS was also inferior (24-month 84.8% vs. 95.5%; log-rank *p* = 0.022; HR 3.08, 95% CI 1.12–8.50) (Figure 2C). When CALLY was analyzed as a continuous variable, each doubling was associated with a 13% reduction in the hazard of a PFS event (HR 0.87, 95% CI 0.76–0.99; *p* = 0.040).

Interim PET was the stronger discriminator. A positive scan was associated with 24-month PFS of 53.3% vs. 92.1% (log-rank *p* < 0.001; HR 5.43, 95% CI 2.55–11.57) and 24-month OS of 83.2% vs. 97.1% (log-rank *p* = 0.004; HR 4.81, 95% CI 1.47–15.77) (Figure 2B,D). An IPS ≥ 3 was prognostic for PFS (HR 2.64, 95% CI 1.30–5.36; *p* = 0.007) but not for OS (HR 2.13, 95% CI 0.79–5.73; *p* = 0.134).

Among the remaining variables, albumin, NLR and IPS ≥3 were significant univariable predictors of PFS, and age, bone marrow involvement and advanced stage were significant univariable predictors of OS (Table 3).

### 3.5. Integrated Modelling

In a multivariable Cox model including CALLY, interim PET, stage and age, only interim PET was independently associated with PFS (HR 4.72, 95% CI 2.17–10.25; *p* < 0.001); CALLY was attenuated to non-significance (HR 1.86, 95% CI 0.79–4.37; *p* = 0.157). Similarly for OS, interim PET was significant (HR 4.18, 95% CI 1.20–14.54; *p* = 0.025) but CALLY was not (Table 4).

The asymmetry was quantified by formal nested comparisons. Adding interim PET to a model that already included CALLY significantly improved fit (PFS χ^2^ = 14.35, *p* < 0.001; OS χ^2^ = 5.02, *p* = 0.025), whereas adding CALLY to a model that already included interim PET did not (PFS χ^2^ = 2.98, *p* = 0.084; OS χ^2^ = 0.56, *p* = 0.456) (Table 4).

Together, the two variables provided an exploratory three-tier stratification, although they were not independent of each other. The 24-month PFS was 94.5% for patients with no adverse factor (n = 87), 79.6% for those with one factor (n = 53), and 41.2% for those with both (n = 17) (overall log-rank *p* < 0.001) (Figure 3A). Compared with the low-risk group, the PFS hazard ratio was 9.48 (95% CI 3.59–25.04; *p* < 0.001) for the high-risk group, whereas the intermediate-risk group did not differ significantly (HR 2.23, 95% CI 0.85–5.81; *p* = 0.102) (Table 4). Median PFS was 12.7 months in the high-risk group and was not reached in the other two groups. Because the high-risk tier contains only 17 patients, with a correspondingly wide confidence interval, and the intermediate tier did not separate significantly from the low-risk tier, this stratification is reported as exploratory and hypothesis-generating rather than as an established clinical model.

### 3.6. Discrimination and Internal Validation

Table 4 shows the optimism-corrected C-indices. CALLY had numerically higher discrimination than the IPS for both endpoints (PFS 0.623 vs. 0.595; OS 0.588 vs. 0.491), and interim PET exceeded both (PFS 0.711; OS 0.688). However, the increment contributed by CALLY over interim PET was small for PFS (0.738 vs. 0.711), absent for OS (0.687 vs. 0.688), and not statistically significant. The four-variable model containing CALLY, interim PET, stage and age had a lower optimism-corrected C-index than the two-variable CALLY + interim PET model (PFS 0.709 vs. 0.738), which requires comment. This is the expected behaviour of optimism correction when the additional predictors carry no independent information: neither stage nor age was significantly associated with PFS in univariable analysis (Table 3) or in the multivariable model (Table 4), so the two extra parameters raised the apparent C-index only marginally while increasing the optimism estimated across bootstrap replications, in which they fit resample-specific noise. The ordering of the corrected values therefore indicates redundancy and incipient overfitting in the larger model rather than a real loss of predictive information, and it is one reason we regard the parsimonious two-variable combination as the more defensible summary of these data.

Time-dependent AUCs at 24 months showed a similar pattern: for PFS, 0.693 for dichotomized CALLY, 0.739 for interim PET and 0.783 for a simple count of the two adverse factors; for OS, 0.685, 0.700 and 0.725, respectively.

### 3.7. Sensitivity Analyses

Restricting the analysis to patients with classical Hodgkin lymphoma who received chemotherapy (n = 175) did not change the results: 24-month PFS was 66.9% vs. 90.9% (*p* = 0.001) and OS 84.8% vs. 95.3% (*p* = 0.015), and the association between CALLY and interim PET was preserved (*p* = 0.017).

Because interim PET is measured after diagnosis, we repeated the survival analysis from a landmark at the interim PET date (median 2.56 months after diagnosis; n = 161). Interim PET remained strongly predictive of both PFS (*p* < 0.001) and OS (*p* = 0.004), and CALLY remained predictive of PFS (*p* = 0.006), although its association with OS was no longer significant in this smaller, later-anchored sample (*p* = 0.170).

Across 2000 bootstrap resamples, the median Youden-optimal cut-off was 1.79 (IQR 1.51–3.30). PFS separation was significant for thresholds from 0.5 to 2.0 (HRs 2.21–3.01) but not at 2.5 or above (Supplementary Table S2). The effect is therefore consistent across a range of low CALLY values, but 1.5 is not a uniquely privileged value.

In an exploratory analysis, CALLY measured at the time of interim PET showed only a trend toward lower values in PET-positive patients (median 10.79 vs. 17.49; *p* = 0.054) and was not significantly associated with PFS (HR 0.85 per doubling; *p* = 0.134). The change in CALLY between diagnosis and interim assessment did not differ by interim PET result (*p* = 0.618).

## 4. Discussion

In 180 patients with newly diagnosed Hodgkin lymphoma, CALLY measured before treatment was associated with interim metabolic response and with both survival endpoints. To our knowledge, this is the first evaluation of CALLY in Hodgkin lymphoma and the first study in any lymphoma to relate it to interim PET. The index has previously been evaluated in hepatocellular carcinoma [18], in a systematic review of solid tumors [19], and in diffuse large B-cell lymphoma [20].

The association between low CALLY and interim PET positivity is biologically plausible, but two levels of argument need to be distinguished: what is established and what remains hypothesis. The established observations are the following. Classical Hodgkin lymphoma is a disease in which a numerically small malignant population shapes an extensive reactive infiltrate, and the composition of that infiltrate predicts outcome [12,13]. Circulating interleukin-6, the principal driver of the hepatic acute-phase response and therefore of CRP, independently predicts treatment failure in this disease [14]; peripheral lymphopenia and a low albumin at diagnosis are associated with adverse outcome and are both components of the IPS [3,16]; and cancer-related systemic inflammation is, more generally, associated with reduced treatment effectiveness [17]. Each of the three CALLY components therefore has an independently documented relationship with outcome, and the composite can be read as a systemic summary of the same process.

What remains a hypothesis is the step from these observations to the interpretation of CALLY as a marker of chemosensitivity. Patients with the most disturbed systemic milieu at diagnosis were the least likely to clear FDG avidity after two cycles (interim PET positivity 29.8% vs. 13.0%), and it is tempting to read this as evidence that the inflammatory microenvironment limits the response of the tumour to chemotherapy. Our data cannot establish that. We measured no cytokines, no tumour-infiltrating lymphocyte subsets, and no other microenvironmental variable, so the proposed chain—microenvironment, systemic inflammatory signature, slow metabolic response—is inferred rather than demonstrated, and a shared upstream cause, in which greater tumour burden produces both the inflammatory signature and the slower response, is equally compatible with what we observed. Cvetković et al. reported the same direction of effect in diffuse large B-cell lymphoma [20], a disease with a far less prominent reactive infiltrate, which argues that the index tracks the host inflammatory state rather than a feature specific to Hodgkin histology; but that is an indirect argument from a different disease. We therefore present the biological account as a plausible hypothesis to be tested with correlative tissue and cytokine studies, not as an established mechanism.

CALLY also showed numerically higher discrimination than the IPS in this cohort, although the two C-indices were not formally compared. Optimism-corrected C-indices favored CALLY for both PFS (0.623 vs. 0.595) and OS (0.588 vs. 0.491), and the IPS was not significantly associated with OS. The loss of prognostic value of the IPS once interim PET is known was first shown by Gallamini et al. [4], and Aygün et al. recently reported that the Glasgow Prognostic Score and the Gustave Roussy Immune Score add limited information beyond the IPS [5]. Other inflammation- and nutrition-based measures have shown limited prognostic value in Hodgkin lymphoma: the hemoglobin–albumin–lymphocyte–platelet score, the composite closest in construction to CALLY, was not associated with survival in 147 patients [31], and the NLR showed no prognostic value in 82 children, in whom early response after two cycles was the independent predictor [32]. The study closest to ours is that of Romano et al., in which NLR and LMR predicted PFS in univariable analysis but only the interim (PET-2) scan remained independent [33], a pattern that mirrors our findings. A practical advantage of CALLY is that it is a single continuous number derived from three routine variables, whereas the IPS requires seven dichotomized inputs. In our cohort, NLR was the only comparator that approached CALLY, and only for PFS (HR 1.05 per unit; *p* = 0.025).

The most important qualification of these findings is that CALLY was not independently prognostic after adjustment for interim PET. Adding CALLY to a model containing interim PET did not significantly improve fit, whereas adding interim PET to a model containing CALLY did. The simplest explanation is that the prognostic information in baseline CALLY is largely mediated by early metabolic response rather than acting in parallel with it: patients who respond slowly tend to have an adverse inflammatory and nutritional profile at diagnosis, and it is the slow response that determines outcome. This mirrors the pattern described by Gallamini et al. for the IPS, whose prognostic significance disappeared once interim PET was known [4], and by Romano et al. for NLR and LMR [33]. Because no formal mediation analysis was performed, the mediation itself remains a hypothesis; what the data establish is the negative result, that CALLY adds no statistically detectable information once the interim scan is known. We therefore do not claim CALLY as an independent prognostic biomarker in Hodgkin lymphoma. It is more accurately described as a pre-treatment correlate—in effect a surrogate—of early metabolic response: informative while response status is still unknown, and largely redundant once the interim scan has been read.

The exploratory analysis of CALLY measured at the time of interim PET bears directly on this interpretation. If the index tracked the biology of response dynamically, the interim value should have separated patients by PFS; it did not (hazard ratio 0.85 per doubling; *p* = 0.134), and the change in CALLY between diagnosis and the interim assessment did not differ by interim PET result (*p* = 0.618). Two readings are possible. The first is that CALLY is informative only as a pre-treatment measurement, because by the interim assessment the index is dominated by treatment effects that move its components in opposing directions for reasons unrelated to disease control: chemotherapy-induced lymphopenia lowers the numerator, while resolution of the acute-phase response lowers the denominator, so the interim value reflects the pharmacology of the regimen at least as much as the behaviour of the lymphoma. The second is that the interim analysis was underpowered, being restricted to patients with paired measurements and to the same small number of events. Either way, the finding constrains the mechanistic proposal, and we have narrowed it accordingly: we claim only that the diagnostic-timepoint measurement, taken before therapy has perturbed any of its three components, is associated with subsequent metabolic response. We do not claim that CALLY is a dynamic marker that can be followed through treatment, and serial measurement is not supported by these data.

The redundancy of CALLY once interim PET is known does not make the index useless, but it does narrow the claim that can be made for it. We accept that the incremental value of CALLY over interim PET is not demonstrable in this cohort: adding CALLY to a model containing interim PET improved fit for neither endpoint, and the gain in optimism-corrected discrimination was 0.027 for PFS and nil for OS. The case for measuring CALLY at baseline therefore does not rest on incremental accuracy over the interim scan. It rests on timing and cost. Interim PET cannot inform the first treatment decision because it does not exist until two cycles have been given—in this cohort the interim scan was performed a median of 2.56 months after diagnosis—whereas CALLY is available on the day of diagnosis, is derived from three laboratory values that are already measured in every patient, and adds neither cost nor radiation exposure. A patient with CALLY < 1.5 had roughly a one-in-three chance of a positive interim scan, compared with about one in eight otherwise. In the interval before the interim scan, where no competing information exists, that difference could reasonably support earlier or closer interim imaging, counseling about the likelihood of treatment escalation, or prioritization for response-adapted trials. Our data do not support using CALLY to select the intensity of initial therapy, and we explicitly caution against this until prospective evidence is available.

The three-tier model is presented as exploratory. It stratified this cohort, although its components are not statistically independent: 24-month PFS was 41.2% with both adverse factors vs. 94.5% with neither, a difference large enough to be clinically meaningful if confirmed. However, the intermediate-risk group did not separate significantly from the low-risk group, and with only 17 patients in the high-risk group, the confidence interval is wide (PFS HR 9.48, 95% CI 3.59–25.04). The model should therefore be regarded as hypothesis-generating, and the language of clinical relevance that appeared in the previous version of the abstract and the results has been removed.

A distinction between statistical and clinical significance should be made explicit here, because the two do not coincide in these results. Statistically, the addition of CALLY to interim PET improved neither model (PFS χ^2^ = 2.98, *p* = 0.084; OS χ^2^ = 0.56, *p* = 0.456). Clinically, the corresponding increments in discrimination—0.027 in the optimism-corrected C-index for PFS and −0.001 for OS—are far below the magnitude that would change a treatment decision and would not reclassify patients across any threshold at which management differs; here the non-significant *p* value and the clinically negligible increment point the same way. The converse also holds. The numerically higher C-index for CALLY than for the IPS (0.623 vs. 0.595 for PFS) was not formally tested and, in any case, describes a difference between two weakly discriminating markers, neither of which is accurate enough to be used alone; it should not be read as clinical superiority. The only comparison in this manuscript that is both statistically robust and clinically consequential is the performance of interim PET itself. We would therefore ask readers not to infer, from a *p* value falling on either side of 0.05, that the corresponding difference either would or would not change practice.

Even with the scales aligned, our threshold of 1.5 is well below those reported in other diseases. Cvetković et al. dichotomized CALLY at 6.5 in diffuse large B-cell lymphoma on the same scale [20], and Iida et al. used 5 in resected hepatocellular carcinoma on the g/dL convention, which corresponds to 50 on our scale [18]; in solid tumors, published cut-offs vary widely [19]. These differences are not a matter of units; they reflect disease biology. Because CRP is in the denominator, the whole distribution shifts with the inflammatory burden of the population studied. In our cohort, the median CRP at diagnosis was 28.7 mg/L (IQR 7.6–91.7); the far higher cut-offs reported in surgically treated cohorts [18,19] can only arise in populations whose CRP is correspondingly lower. The cytokine-driven acute-phase response that characterizes untreated Hodgkin lymphoma [14] therefore compresses CALLY into a low range: the diffuse large B-cell lymphoma threshold would classify most of our patients as high risk, and the hepatocellular carcinoma threshold, which lies far above our upper quartile of 10.32, almost all of them. This ordering—Hodgkin lymphoma below diffuse large B-cell lymphoma, and both far below resectable solid tumors—follows inflammatory burden rather than any intrinsic property of the index. Thresholds for any composite measure with an inflammatory denominator therefore need to be recalibrated for each disease.

This study has several limitations. It was retrospective and conducted at a single center. The radiotherapy rate of 5.6% reflects an institutional preference for chemotherapy alone in patients achieving complete metabolic response, and centres that consolidate with radiotherapy more often may not reproduce this outcome distribution. Several subgroups were small: only 9 patients had stage I disease, which is why stage was analysed throughout as early versus advanced rather than by individual Ann Arbor category, and the high-risk tier of the integrated model contains only 17 patients, with a correspondingly wide confidence interval, so estimates in these strata are exploratory. Events were few (32 PFS events and 16 deaths), which limits the power of the multivariable models: the non-significant likelihood ratio test for CALLY added to interim PET (*p* = 0.084 for PFS) is compatible both with a truly absent effect and with a real but small effect that 27 events cannot resolve. The cut-off of 1.5 was derived from these data rather than prespecified. Anchoring it to interim PET rather than to survival avoids circularity in the survival analyses, and bootstrap analysis showed a consistent effect across thresholds from 0.5 to 2.0, but the specific value remains optimistic and requires external validation [26]. For the reasons set out above, it is also specific to the scale used here and to a population with the inflammatory burden of untreated Hodgkin lymphoma, and it should not be transferred to or from surgical cohorts. The median follow-up of 30.5 months is short for a disease in which late relapses occur. Interim PET was scored in routine practice without central review; although expert readers agree well on the binary Deauville 1–3 vs. 4–5 distinction [34], inter-reader concordance is poorest for the intermediate scores and lowest of all for a score of 3, which is adjacent to the 3/4 boundary that defines positivity here [35]. Finally, the cohort was too small for analyses stratified by treatment, and treatment was heterogeneous, with a minority of patients receiving brentuximab vedotin–AVD.

These limitations bear directly on external validity, which deserves a more explicit statement than we gave previously. The cohort comes from a single tertiary centre with an institutional treatment pattern—ABVD in the large majority and radiotherapy in 5.6%—that will not match practice elsewhere, and the distribution of CALLY is itself set by the inflammatory burden of the population sampled, so both the threshold and the observed event rates are properties of this cohort as much as of the index. A follow-up of 30.5 months is too short to capture late relapse, and 32 progression-free survival events and 16 deaths are too few to support a model intended for assigning risk to individual patients. Most importantly, frontline treatment of Hodgkin lymphoma is changing: brentuximab vedotin–AVD and, more recently, nivolumab–AVD have displaced ABVD as first-line therapy in advanced-stage disease [23,26]. Neither trial was response-adapted, so the prognostic weight of interim PET under these regimens has not been established, and a baseline marker of host immune competence may well behave differently under checkpoint blockade than under ABVD. Translating these findings into practice would therefore require a prospective, multicentre study using contemporary protocols, with central Deauville review and a prespecified threshold, powered on a number of events an order of magnitude larger than ours. Until such data exist, the CALLY index and the integrated model described here should be regarded as research tools; we do not recommend either for routine clinical use, and specifically not for selecting the intensity of initial therapy.

Formal testing of the mediation hypothesis would require an independent cohort with central Deauville review and on the order of 100 progression events, rather than the 32 available here. The same cohort would allow the question raised above—whether CALLY retains any value under brentuximab vedotin and checkpoint inhibitor based frontline regimens [23,36]—to be answered directly.

## 5. Conclusions

Baseline CALLY < 1.5 identified patients with a 29.8% rate of interim PET positivity, compared with 13.0% above the threshold, and a 24-month progression-free survival of 66.9% vs. 89.8%. The index showed numerically higher discrimination than the International Prognostic Score for both endpoints (optimism-corrected C-index 0.623 vs. 0.595 for progression-free survival; the difference was not formally tested) but was not independently prognostic once interim PET was available. CALLY can therefore identify, on the day of diagnosis and from three routinely obtained laboratory values, patients who are likely to respond slowly, and it may serve as an adjunct to, rather than a replacement for, interim response assessment. Discrimination was modest throughout, the threshold was derived within this cohort, and the three-tier model is exploratory; prospective multicentre validation in contemporary treatment protocols is required before the index is considered for routine practice.

## Figures and Tables

**Figure 1:**
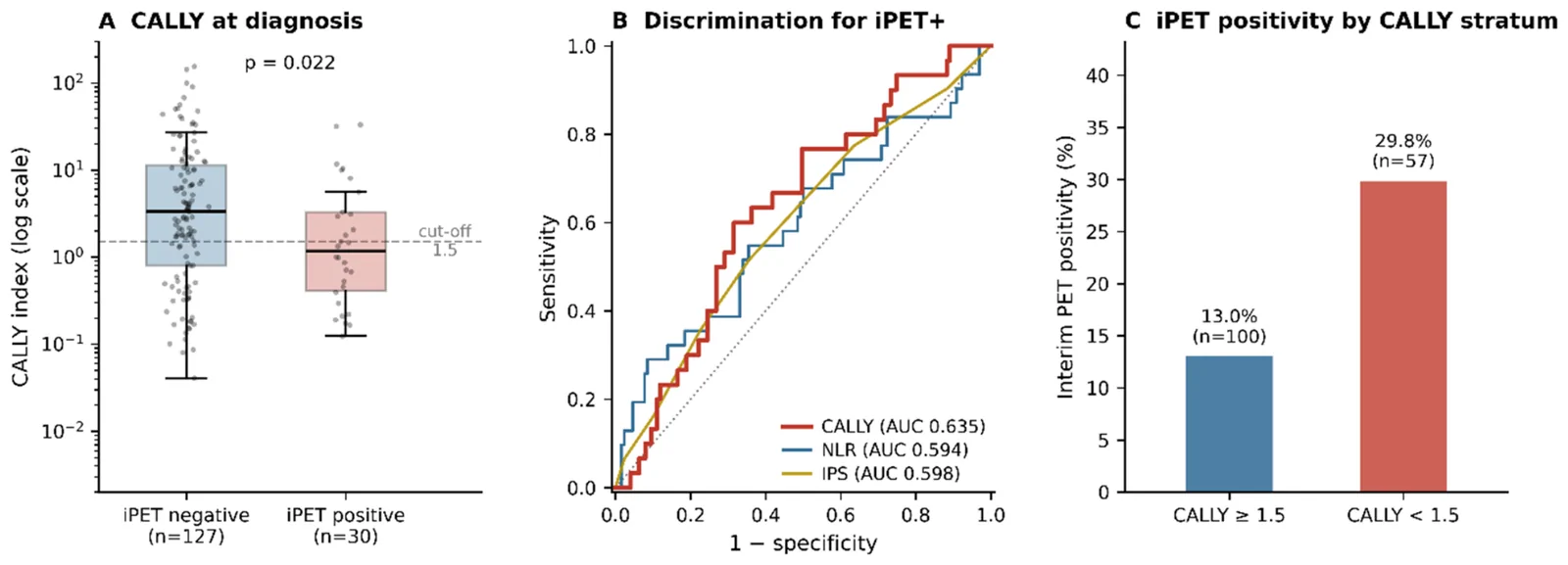
Baseline CALLY index and interim PET response. (**A**) Distribution of baseline CALLY by interim PET result, plotted on a logarithmic scale; boxes show median and interquartile range, whiskers extend to 1.5 × IQR, and individual patients are overlaid. The dashed line marks the 1.5 threshold. (**B**) Receiver operating characteristic curves for predicting interim PET positivity, comparing CALLY with NLR and the IPS. (**C**) Proportion of patients with a positive interim PET, by CALLY stratum. iPET, interim PET; NLR, neutrophil-to-lymphocyte ratio; IPS, International Prognostic Score; AUC, area under the curve.

**Figure 2:**
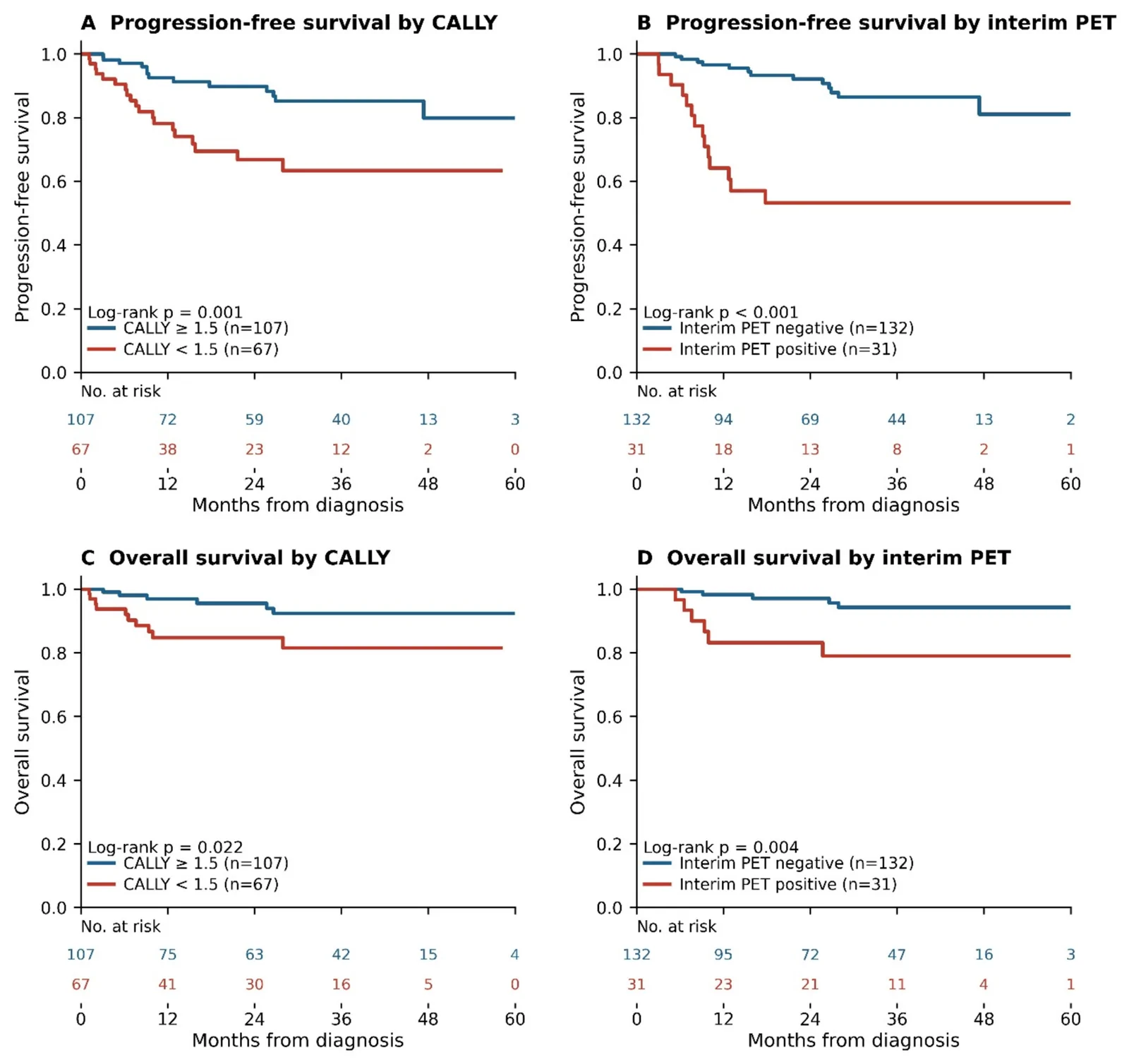
Survival by CALLY index and by interim PET response. Kaplan–Meier estimates of progression-free survival (**A**,**B**) and overall survival (**C**,**D**), stratified by baseline CALLY index (**A**,**C**) and by interim PET result (**B**,**D**). Numbers at risk are shown below each panel. *p* values are from the log-rank test.

**Figure 3:**
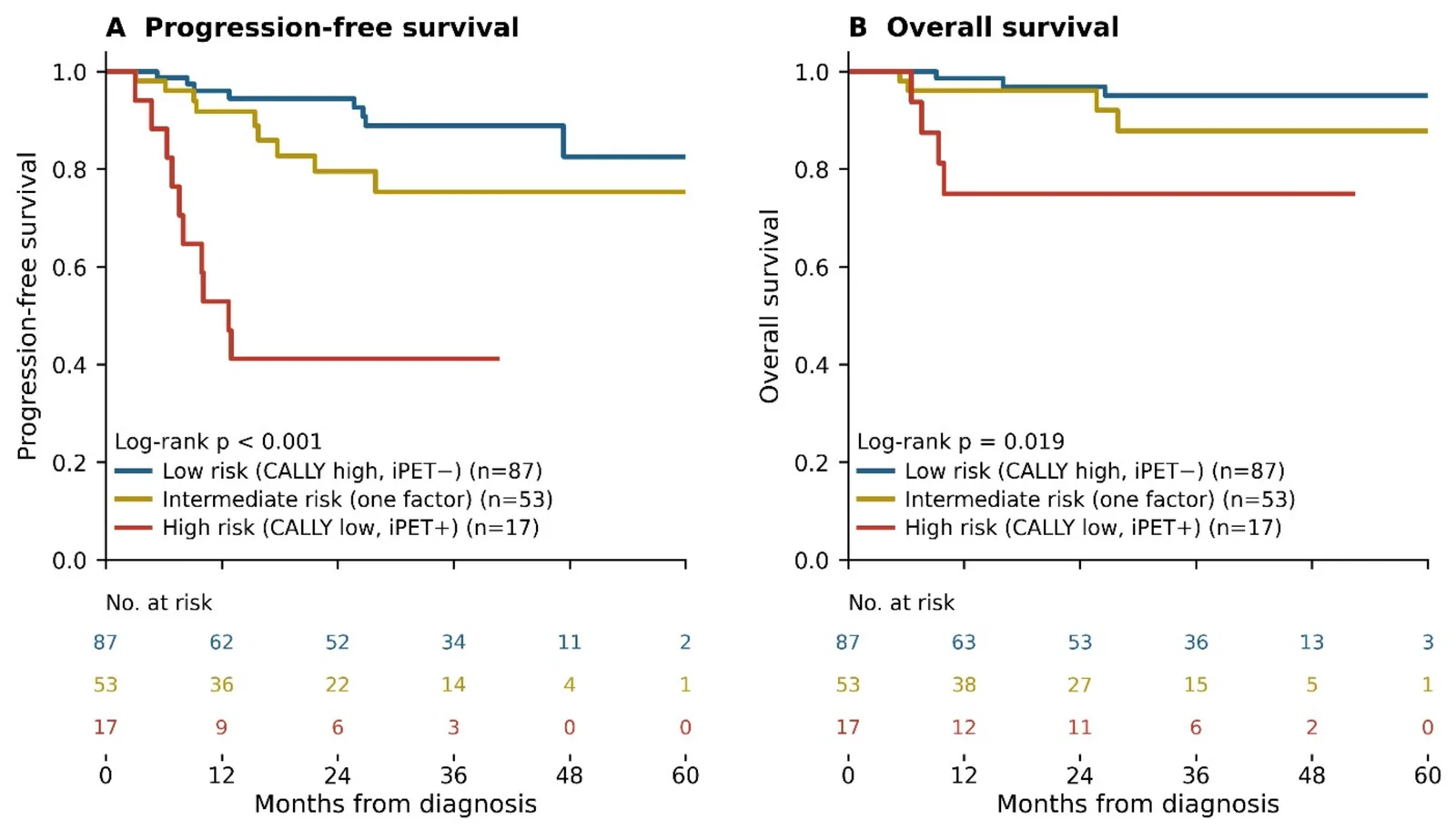
Integrated CALLY–PET risk model. Kaplan–Meier estimates of progression-free survival (**A**) and overall survival (**B**) for the three-tier model. Low risk: CALLY ≥ 1.5 and interim PET negative. Intermediate risk: exactly one adverse factor. High risk: CALLY < 1.5 and interim PET positive.

**Table 1:** Baseline characteristics of the study cohort (n = 180).

Characteristic	Value
Age, years, median (IQR)	34 (25–51)
Male sex, n (%)	112 (62.2)
**Histology, n (%)**	
Nodular sclerosis	96 (53.3)
Mixed cellularity	62 (34.4)
Classical, not otherwise specified	13 (7.2)
Nodular lymphocyte-predominant	5 (2.8)
Lymphocyte-rich	3 (1.7)
Lymphocyte-depleted	1 (0.6)
**Ann Arbor stage, n (%)**	
I	9 (5.0)
II	73 (40.8)
III	30 (16.8)
IV	67 (37.4)
Advanced stage (III–IV), n/N (%)	97/179 (54.2)
B symptoms, n (%)	92 (51.1)
Bulky disease, n/N (%)	41/179 (22.9)
Extranodal involvement, n/N (%)	66/179 (36.9)
Bone marrow involvement, n/N (%)	54/179 (30.2)
IPS ≥ 3, n/N (%)	71/176 (40.3)
Radiotherapy, n/N (%)	10/178 (5.6)
Autologous stem cell transplantation, n/N (%)	10/161 (6.2)
**Laboratory at diagnosis, median (IQR)**	
Haemoglobin, g/dL	12.0 (10.0–13.5)
White blood cells, /µL	8995 (6685–12,243)
Lymphocytes, /µL	1645 (1078–2178)
Albumin, g/dL	4.15 (3.70–4.50)
C-reactive protein, mg/L	28.7 (7.6–91.7)
Lactate dehydrogenase, U/L	214 (177–259)
Erythrocyte sedimentation rate, mm/h	41.5 (17.0–64.0)
NLR	4.19 (2.71–6.46)
PLR	228.5 (152.2–319.9)
LMR	1.99 (1.26–3.05)
CALLY index	2.50 (0.50–10.32)
**Outcomes**	
Median follow-up (reverse KM), months	30.5
Interim PET positive, n/N (%)	31/163 (19.0)
End-of-treatment complete metabolic response, n/N (%)	127/146 (87.0)
Progression-free survival events, n	32
Deaths, n	16

IQR, interquartile range; IPS, International Prognostic Score; NLR, neutrophil-to-lymphocyte ratio; PLR, platelet-to-lymphocyte ratio; LMR, lymphocyte-to-monocyte ratio; KM, Kaplan–Meier. Laboratory values are given in clinical reporting units; the CALLY index is calculated from albumin in g/L and lymphocytes in ×10^9^/L (Section 2.3), so albumin must be multiplied by 10 and the lymphocyte count divided by 1000 before the formula is applied.

**Table 2:** Baseline characteristics by CALLY stratum.

Variable	CALLY < 1.5 (n = 67)	CALLY ≥ 1.5 (n = 107)	*p*
Age, years	36.0 (24.5–50.5)	34.0 (25.0–51.0)	0.957
Male sex, n (%)	44 (65.7)	65 (60.7)	0.525
Haemoglobin, g/dL	9.7 (8.6–11.9)	12.8 (11.6–14.6)	<0.001
White blood cells, /µL	10,380 (6295–15,680)	8670 (6940–10,740)	0.061
Lymphocytes, /µL	1150 (630–1830)	1800 (1360–2280)	<0.001
Albumin, g/dL	3.6 (3.2–4.0)	4.4 (4.1–4.6)	<0.001
C-reactive protein, mg/L	123.0 (64.7–177.0)	10.4 (4.4–23.9)	<0.001
Lactate dehydrogenase, U/L	235 (192–284)	203 (173–255)	0.015
Erythrocyte sedimentation rate, mm/h	66 (45–80)	23 (12–47)	<0.001
NLR	6.6 (4.3–10.6)	3.3 (2.0–4.9)	<0.001
IPS, points	4.0 (3.0–5.0)	1.5 (1.0–2.0)	<0.001
B symptoms, n/N (%)	50/67 (74.6)	39/107 (36.4)	<0.001
Bulky disease, n/N (%)	22/67 (32.8)	18/106 (17.0)	0.025
Extranodal involvement, n/N (%)	39/67 (58.2)	26/106 (24.5)	<0.001
Bone marrow involvement, n/N (%)	32/67 (47.8)	19/106 (17.9)	<0.001
Advanced stage (III–IV), n/N (%)	48/67 (71.6)	45/106 (42.5)	<0.001
IPS ≥ 3, n/N (%)	53/67 (79.1)	17/106 (16.0)	<0.001
Interim PET positive, n/N (%)	17/57 (29.8)	13/100 (13.0)	0.012
End-of-treatment complete response, n/N (%)	40/50 (80.0)	82/90 (91.1)	0.070
Autologous stem cell transplantation, n/N (%)	7/59 (11.9)	3/97 (3.1)	0.043

Continuous variables are median (IQR), compared by Mann–Whitney U test; categorical variables compared by Fisher’s exact test.

**Table 3:** Univariable predictors of interim PET positivity and of survival.

(**a**) **Interim PET positivity—logistic regression and discrimination**							
**Variable**		**OR (95% CI)**			***p***	**AUC**	
CALLY < 1.5		2.84 (1.26–6.42)			0.012	0.635	
Extranodal involvement		2.87 (1.29–6.38)			0.010	—	
Bulky disease		2.14 (0.92–5.00)			0.079	—	
IPS ≥ 3		1.92 (0.87–4.25)			0.105	0.598	
B symptoms		1.73 (0.78–3.86)			0.177	—	
Advanced stage (III–IV)		1.14 (0.52–2.51)			0.739	—	
Age (per year)		0.99 (0.96–1.02)			0.414	—	
CRP (continuous)		—			0.008	0.655	
NLR (continuous)		—			0.104	0.594	
Albumin (continuous)		—			0.314	0.558	
LMR (continuous)		—			0.695	0.523	
PLR (continuous)		—			0.976	0.502	
(**b**) **Univariable Cox models**							
**Variable**	**PFS HR (95% CI)**		***p***	**OS HR (95% CI)**			***p***
Interim PET positive	5.43 (2.55–11.57)		<0.001	4.81 (1.47–15.77)			0.010
CALLY < 1.5	3.01 (1.48–6.14)		0.002	3.08 (1.12–8.50)			0.029
IPS ≥ 3	2.64 (1.30–5.36)		0.007	2.13 (0.79–5.73)			0.134
Advanced stage (III–IV)	2.06 (0.99–4.30)		0.053	3.16 (1.02–9.84)			0.047
B symptoms	1.89 (0.91–3.92)		0.088	2.12 (0.74–6.11)			0.163
Bone marrow involvement	1.78 (0.87–3.64)		0.117	3.84 (1.42–10.35)			0.008
Extranodal involvement	1.26 (0.62–2.55)		0.524	1.79 (0.67–4.76)			0.247
Bulky disease	1.20 (0.52–2.79)		0.669	0.58 (0.13–2.55)			0.468
Age (per year)	1.02 (0.99–1.04)		0.151	1.04 (1.01–1.08)			0.005
Albumin (per g/dL)	0.47 (0.29–0.78)		0.003	0.60 (0.29–1.22)			0.160
NLR (per unit)	1.05 (1.01–1.10)		0.025	1.02 (0.95–1.10)			0.521
LMR (per unit)	0.94 (0.72–1.22)		0.645	0.83 (0.55–1.25)			0.379
CALLY (per doubling)	0.87 (0.76–0.99)		0.040	0.89 (0.74–1.07)			0.210

HR, hazard ratio; OR, odds ratio; AUC, area under the receiver operating characteristic curve.

**Table 4:** Multivariable models and model discrimination.

(**a**) **Multivariable Cox models (n = 157)**											
**Variable**	**PFS HR (95% CI)**					***p***		**OS HR (95% CI)**			***p***
Interim PET positive	4.72 (2.17–10.25)					<0.001		4.18 (1.20–14.54)			0.025
CALLY < 1.5	1.86 (0.79–4.37)					0.157		1.34 (0.35–5.12)			0.674
Advanced stage (III–IV)	1.20 (0.52–2.77)					0.675		1.66 (0.44–6.31)			0.456
Age (per year)	1.00 (0.97–1.02)					0.721		1.01 (0.98–1.05)			0.492
PFS events = 27; OS events = 11. No variable violated the proportional hazards assumption (all *p* > 0.05).											
(**b**) **Discrimination (Harrell C-index, optimism-corrected, 500 bootstrap replications)**											
**Model**							**PFS**			**OS**	
IPS ≥ 3							0.595			0.491	
CALLY < 1.5							0.623			0.588	
CALLY (continuous, log_2_)							0.582			0.500	
Interim PET alone							0.711			0.688	
CALLY + interim PET							0.738			0.687	
IPS + interim PET							0.740			0.662	
CALLY + interim PET + stage + age							0.709			0.683	
(**c**) **Nested likelihood ratio tests**											
**Comparison**				**PFS χ^2^ (*p*)**					**OS χ^2^ (*p*)**		
Interim PET added to CALLY				14.35 (<0.001)					5.02 (0.025)		
CALLY added to interim PET				2.98 (0.084)					0.56 (0.456)		
CALLY added to IPS				2.37 (0.124)					1.83 (0.176)		
(**d**) **Integrated three-tier risk model**											
**Risk group**		**n**	**24-month PFS**		**24-month OS**			**PFS HR vs. low risk (95% CI)**			***p***
Low (CALLY ≥ 1.5 and interim PET negative)		87	94.5%		96.9%			reference			—
Intermediate (one adverse factor)		53	79.6%		96.1%			2.23 (0.85–5.81)			0.102
High (CALLY < 1.5 and interim PET positive)		17	41.2%		75.0%			9.48 (3.59–25.04)			<0.001

Overall log-rank *p* < 0.001 for PFS and *p* = 0.019 for OS. CI, confidence interval; HR, hazard ratio; OS, overall survival; PFS, progression-free survival. The concordance index is Harrell’s C, corrected for optimism by 500 bootstrap replications. Likelihood ratio tests compare nested Cox models fitted to the same patients.

## Data Availability

The raw data supporting the conclusions of this article will be made available by the authors on request.

## Supplementary Materials

The following supporting information can be downloaded at: https://www.mdpi.com/article/10.3390/cancers18193205/s1, Figure S1: Patient flow diagram; Table S1: Landmark analysis anchored at the interim PET date; Table S2: Cut-off stability: bootstrap distribution of the Youden threshold and survival separation across alternative thresholds; Table S3: Extent of missing data for key variables; Table S4: Time-dependent discrimination at 24 months; Table S5: Exploratory analysis of CALLY at the interim timepoint; File S1: Completed STROBE checklist for cohort studies.

## Abbreviations

The following abbreviations are used in this manuscript:

ABVD	doxorubicin–bleomycin–vinblastine–dacarbazine
AUC	area under the receiver operating characteristic curve
CALLY	C-reactive protein–albumin–lymphocyte
CI	confidence interval
CRP	C-reactive protein
ESR	erythrocyte sedimentation rate
^18^F-FDG	^18^F-fluorodeoxyglucose
HR	hazard ratio
IPS	International Prognostic Score
IQR	interquartile range
LMR	lymphocyte-to-monocyte ratio
NLR	neutrophil-to-lymphocyte ratio
OR	odds ratio
OS	overall survival
PET/CT	positron emission tomography/computed tomography
PFS	progression-free survival
PLR	platelet-to-lymphocyte ratio
