# Supplementary material for: Integrated Prognostic Modelling with the CALLY Index and Interim PET/CT in Hodgkin Lymphoma
Source: Cancers (Basel). 2026 Oct 4;18(19):3205. doi: 10.3390/cancers18193205 (PMC13664377; doi:10.3390/cancers18193205)
Supplement: Supplementary file 1 [file cancers-18-03205-s001.zip › cancers-4547279-Supplementary File S1 - STROBE.pdf]

Supplementary File S1. Completed STROBE checklist for cohort studies

Manuscript: Integrated Prognostic Modelling with the CALLY Index and Interim PET/CT in Hodgkin Lymphoma

#	Item	Recommendation	Reported in
1a	Title and abstract	Indicate the study's design in the title or abstract	Abstract, Methods sentence ("We performed a retrospective study of 180 consecutive patients")
1b		Provide an informative and balanced summary in the abstract	Abstract (structured: Objective, Methods, Results, Conclusion)
2	Background/rationale	Explain the scientific background and rationale	Introduction, paragraphs 1–5
3	Objectives	State specific objectives, including any prespecified hypotheses	Introduction, final paragraph (three stated questions)
4	Study design	Present key elements of study design early in the paper	Methods 2.1
5	Setting	Describe setting, locations, and relevant dates	Methods 2.1 (single tertiary haematology centre); Results 3.1 (median follow-up 30.5 months)
6a	Participants	Give eligibility criteria, and the sources and methods of selection	Methods 2.1; Supplementary Figure S1
6b		For matched studies, give matching criteria	Not applicable (unmatched cohort)
7	Variables	Clearly define all outcomes, exposures, predictors, potential confounders and effect modifiers	Methods 2.2 (PET response), 2.3 (CALLY, comparators), 2.4 (endpoints)
8	Data sources / measurement	For each variable, give sources of data and details of methods of assessment	Methods 2.2, 2.3; Supplementary Methods (unit handling)
9	Bias	Describe any efforts to address potential sources of bias	Methods 2.5 (threshold derived from interim PET rather than survival, to avoid circularity; landmark analysis for immortal-time bias)
10	Study size	Explain how the study size was arrived at	Methods 2.1 (all consecutive eligible patients; no a priori size calculation); Discussion (limitation)
11	Quantitative variables	Explain how quantitative variables were handled; describe groupings and why	Methods 2.5, subsection "Derivation of the CALLY threshold"; Supplementary Table S2

12a	Statistical methods	Describe all statistical methods, including those used to control for confounding	Methods 2.5
12b		Describe any methods used to examine subgroups and interactions	Results 3.7 (classical HL only; landmark)
12c		Explain how missing data were addressed	Methods 2.5 (complete cases); Supplementary Table S3
12d		If applicable, explain how loss to follow-up was addressed	Methods 2.4 (censoring at last contact); Results 3.1 (reverse Kaplan–Meier follow-up)
12e		Describe any sensitivity analyses	Results 3.7; Methods 2.5 (threshold grid, bootstrap)
13a	Participants	Report numbers of individuals at each stage of the study	Methods 2.1; Supplementary Figure S1
13b		Give reasons for non-participation at each stage	Methods 2.1 (two missing dates/vital status, one non-positive follow-up interval)
13c		Consider use of a flow diagram	Supplementary Figure S1
14a	Descriptive data	Give characteristics of study participants and information on exposures and potential confounders	Table 1; Results 3.1
14b		Indicate number of participants with missing data for each variable of interest	Supplementary Table S3
14c		Summarise follow-up time	Results 3.1 (median 30.5 months, reverse Kaplan–Meier)
15	Outcome data	Report numbers of outcome events or summary measures over time	Results 3.1 (32 PFS events, 16 deaths); Tables 3, 4
16a	Main results	Give unadjusted estimates and, if applicable, confounder-adjusted estimates and their precision	Table 3 (univariable), Table 4 (multivariable), all with 95% confidence intervals
16b		Report category boundaries when continuous variables were categorised	Methods 2.5 (CALLY < 1.5, with derivation and rounding effect); Methods 2.3 (IPS ≥ 3)
16c		If relevant, consider translating estimates of relative risk into absolute risk	Results 3.4, 3.5 (24- and 36-month survival percentages); Discussion (one-in-three versus one-in-eight)
17	Other analyses	Report other analyses done	Results 3.6 (discrimination, internal validation), 3.7 (sensitivity analyses)
18	Key results	Summarise key results with reference to study objectives	Discussion, paragraph 1; Conclusion

19	Limitations	Discuss limitations, taking into account sources of potential bias or imprecision	Discussion, limitations paragraph
20	Interpretation	Give a cautious overall interpretation considering objectives, limitations, multiplicity of analyses, results from similar studies, and other relevant evidence	Discussion, paragraphs 2–7
21	Generalisability	Discuss the generalisability (external validity) of the study results	Discussion (single centre, radiotherapy rate, threshold specific to scale and population)
22	Funding	Give the source of funding and the role of the funders	Declarations, Funding

Note. An Explanation and Elaboration article discusses each checklist item and gives methodological background and published examples of transparent reporting. The STROBE checklist is best used in conjunction with that article, freely available at https://www.strobe-statement.org.
