# Supplementary material for: Integrated Prognostic Modelling with the CALLY Index and Interim PET/CT in Hodgkin Lymphoma
Source: Cancers (Basel). 2026 Oct 4;18(19):3205. doi: 10.3390/cancers18193205 (PMC13664377; doi:10.3390/cancers18193205)
Supplement: Supplementary file 1 [file cancers-18-03205-s001.zip › cancers-4547279-supplementary.pdf]

Supplementary material

- **Supplementary Figure S1.** Patient flow diagram.
- **Supplementary Table S1.** Landmark analysis anchored at the interim PET date.
- **Supplementary Table S2.** Cut-off stability: bootstrap distribution of the Youden threshold and survival separation across alternative thresholds.
- **Supplementary Table S3.** Extent of missing data for key variables.
- **Supplementary Table S4.** Time-dependent discrimination at 24 months.
- **Supplementary Table S5.** Exploratory analysis of CALLY at the interim timepoint.
- **Supplementary File S1.** Completed STROBE checklist for cohort studies.

Supplementary Figure S1. Patient flow diagram

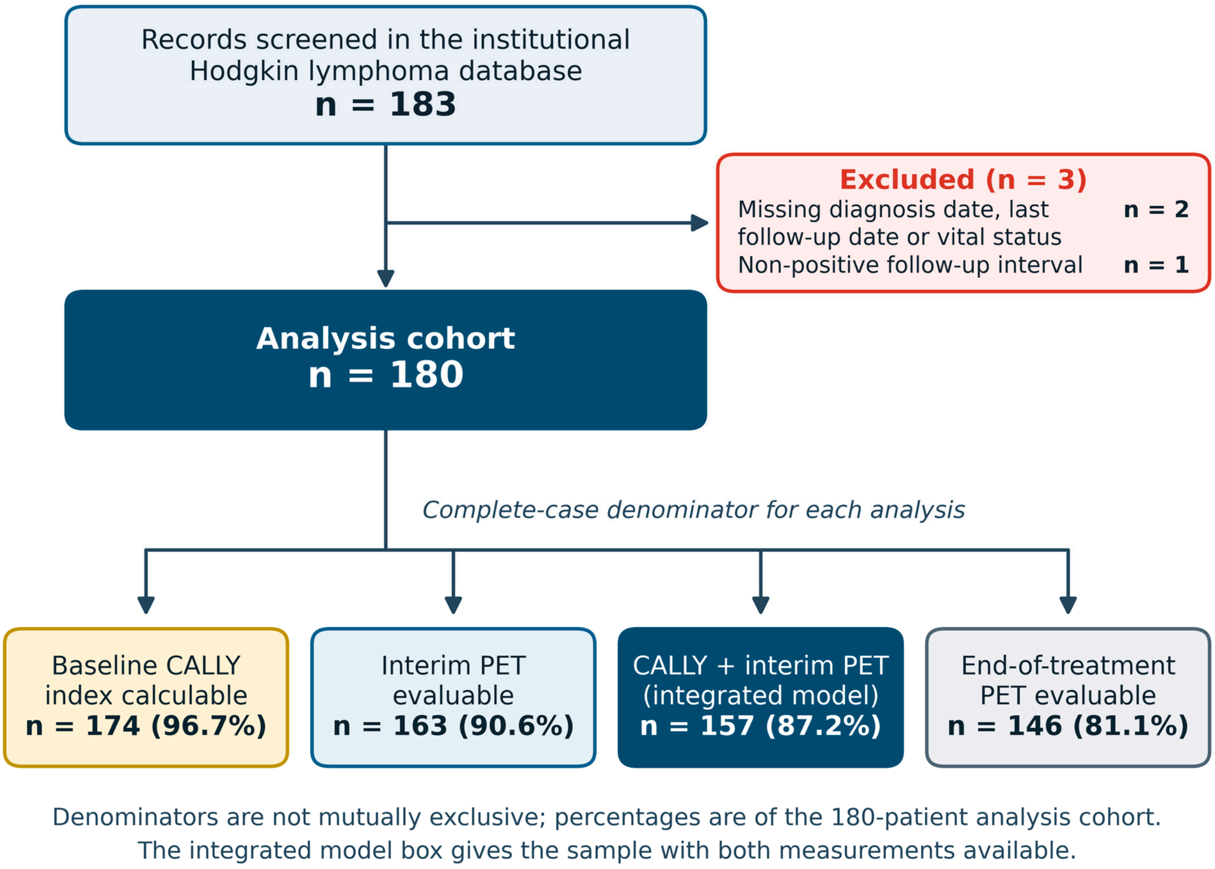

Consecutive patients aged ≥18 years with histologically confirmed Hodgkin lymphoma were identified from the institutional database. Of 183 screened records, 2 lacked a diagnosis date, last follow-up date or vital status and 1 had a non-positive follow-up interval, leaving 180 patients. The four boxes below the analysis cohort give the complete-case sample size available for each analysis; they are not mutually exclusive.

Supplementary Table S1. Landmark analysis anchored at the interim PET date

To exclude immortal-time bias, survival was re-analysed from the date of interim PET rather than from diagnosis. Patients with an interim PET performed within 12 months of diagnosis and who were event-free at that landmark were included (n = 161; median interval from diagnosis to interim PET 2.56 months, IQR 2.07–3.19).

(a) Progression-free survival from the landmark (n = 160, 26 events)

Stratum	n	Events	24-month PFS	36-month PFS	Log-rank p
Interim PET negative	130	13	89.1%	86.2%	< 0.001
Interim PET positive	30	13	55.3%	55.3%	
CALLY ≥ 1.5	98	11	88.1%	86.5%	0.006
CALLY < 1.5	56	15	70.0%	66.1%	

(b) Overall survival from the landmark (n = 161, 11 events)

Stratum	n	Events	24-month OS	36-month OS	Log-rank p
Interim PET negative	130	5	95.8%	94.3%	0.004
Interim PET positive	31	6	78.7%	78.7%	
CALLY ≥ 1.5	99	5	93.1%	93.1%	0.170
CALLY < 1.5	56	6	90.3%	86.7%	

Interim PET retained strong prognostic value for both endpoints after landmarking. CALLY remained prognostic for PFS but lost significance for OS in this smaller, later-anchored sample. Strata do not sum to the same total across panels because CALLY was not calculable in every landmarked patient.

PFS, progression-free survival; OS, overall survival; PET, positron emission tomography.

Supplementary Table S2. Cut-off stability: bootstrap distribution of the Youden threshold and survival separation across alternative thresholds

(a) Bootstrap distribution of the Youden-optimal threshold (2,000 resamples)

Statistic	Value
Median bootstrap cut-off	1.79
Interquartile range	1.51–3.30
95% interval	0.98–11.75
Proportion of resampled cut-offs within 0.5–4.0	92.3%

(b) Progression-free survival separation across alternative thresholds (n = 174)

Threshold	n below threshold	HR (CALLY low vs high)	Log-rank p
0.5	44	2.62	0.006
1.0	58	2.49	0.008
**1.5**	**67**	**3.01**	**0.001**
2.0	81	2.21	0.027
2.5	87	1.97	0.060
5.0	111	1.62	0.214

The prognostic separation is significant across thresholds from 0.5 to 2.0 and disappears at 2.5 and above. The 1.5 value maximises separation in this dataset but is not uniquely privileged, and carries the optimism inherent to data-derived cut-offs.

HR, hazard ratio. Thresholds are on the scale defined in Supplementary Methods; on the g/dL convention each value should be divided by 10.

Supplementary Table S3. Extent of missing data for key variables

Variable	Missing, n/180	%
Albumin at diagnosis	2	1.1
Lymphocyte count at diagnosis	2	1.1
Ann Arbor stage	1	0.6
International Prognostic Score	4	2.2
C-reactive protein at diagnosis	6	3.3
**Baseline CALLY index**	**6**	**3.3**
**Interim PET result**	**17**	**9.4**
CALLY at interim timepoint	25	13.9
End-of-treatment PET result	34	18.9

All analyses used complete cases. No imputation was performed. The CALLY index is missing whenever any of its three components is missing, so its missingness is not the sum of the component rows.

Supplementary Table S4. Time-dependent discrimination at 24 months

Case/dynamic-control area under the curve, comparing patients with an event within 24 months against those event-free and still under observation beyond 24 months.

Marker	PFS AUC	OS AUC
CALLY (continuous)	0.646	0.630
CALLY < 1.5	0.693	0.685
Interim PET	0.739	0.700
IPS (continuous)	0.718	0.704
IPS ≥ 3	0.662	0.636
NLR (continuous)	0.653	0.617
CALLY + interim PET (0–2 adverse factors)	**0.783**	**0.725**

Evaluable: 111 patients (27 PFS events) and 108 patients (13 deaths) at the 24-month horizon.

AUC, area under the receiver operating characteristic curve; IPS, International Prognostic Score; NLR, neutrophil-to-lymphocyte ratio.

Supplementary Table S5. Exploratory analysis of CALLY at the interim timepoint

Analysis	Result
Interim CALLY, interim PET positive vs negative (median)	10.79 vs 17.49; $p = 0.054$ ($n = 155$)
Interim CALLY and PFS, per doubling	HR 0.85 (95% CI 0.69–1.05); $p = 0.134$
Change in $\log_2$ CALLY (interim − baseline), PET positive vs negative	2.33 vs 2.26; $p = 0.618$ ($n = 150$)

CALLY rose substantially in both groups between diagnosis and the interim assessment, consistent with resolution of the acute-phase response under effective cytoreduction, but the magnitude of that rise did not distinguish responders from non-responders. The prognostic signal in this cohort resides in the baseline measurement, not in its change.

These analyses are exploratory and were not corrected for multiple comparisons.
